# Correspondence

**Published:** 1859-02

**Authors:** 


*Correspondence.*—We received last month the following interesting letters, but they were unfortunately crowded out of our last issue. We with pleasure, give them a place in this number:

CONNEAUT, Ohio, Dec. 16th, 1858.

DR. FLINT:

Dear Sir,—Allow me to present to your notice the following report of a case of malformation occurring in my practice. It struck me with peculiar interest, inasmuch as I had never heard of a like case. If you deem it of sufficient interest, you may give it an insertion in your valuable journal.

The case is that of a third child of healthy parents; labor natural, of about ten hours duration. At birth, it uttered a feeble cry, and required constant effort, on our part, to continue the respiration, so feebly begun. I allowed the cord to remain untied, until pulsation ceased at the placental extremity, using the ordinary means resorted to in cases of imperfect expansion of the lungs, with, however, quite unsatisfactory results; the child began to moan, which it continued to do, "except at short intervals," while it lived. It frequently become quite livid, as in cyanosis, with intervals of natural color. The action of the heart was rather that of palpitation, or labored beating; the pulse was scarcely perceptible at the wrist; bladder was evacuated two or three times; no evacuation of the bowels. The child continued to live, with but slight deviations from the above history, for about eighteen hours, when, in slight convulsions, it died. The fact that this was the third child of the same parents that presented the same train of symptoms, (the first surviving nine, the second five, and the subject of this report but eighteen hours,) led me to ask for a *post-mortem* examination, which was heartily assented to. Consequently, about twenty-eight hours after death, assisted by my friend, Dr. Fifield, of this place, we proceeded to the examination. The whole surface of the body, with but slight exceptions, was quite livid with capillary congestion, but chiefly so upon the depending portions. On opening into the cavity of the thorax, the parts appeared in natural position; but on attempting to remove the contents of the cavity, we discovered the pericardium to be adherent to the diaphragm, over a circular space, at least one and a half inches in diameter; a silver half dollar would not cover the space upon the diaphragm, from which the pericardium was carefully dissected off. It contained a more than normal quantity of slightly colored serum, as did also the right pleural cavity. The greater part of the lungs had, the appearance of liver, and only in small portions did they crepitate, or give evidence of having been expanded. Upon inflating them, however, they appeared quite healthy. The heart was filled with firm clots. The ductus artereosus, and the foramen ovale were quite open, exhibiting no signs of contracting.

Query? May we infer from this that the same malformation existed in the two previous cases?

Very respectfully, yours,

E. D. Merriam.

---

Waterford, Erie Co., Penn., Dec. 11th, 1858.

Austin Flint, Jr., M. D.:

My Dear Sir,—Enclosed you will find two pieces of glass, and a brief history of the case connected with it. The glass was in one piece when removed from the arm, but has been broken accidentally since. It being a case rather peculiar, you are at liberty to publish it if you think proper.

I was called to see Mrs. A., the wife of a hotel-keeper of this place, aged about 40; found her complaining of a difficulty to use her right arm, accompanied with some pain, just above the elbow joint, on the outside of the arm; said she thought she could feel a piece of bone loose in her arm. On examining it closely, I found a little tenderness, slight swelling, and was sure I could feel some foreign body, by pressing pretty hard upon the parts. I requested the lady to let me make an opening, but as she objected, I ordered a poultice, and the next day found her much the same as when I left her; all the symptoms had become a little aggravated. I then insisted on making an opening, and the lady finally consented. I made an incision one inch in length, and one-half inch deep. I then introduced my finger, and was certain I was near to a foreign substance. I then made my incision a little deeper, and with a pair of small forceps, I extracted a piece of common window glass, a little more than one inch in length, and one-fourth of an inch in width at one end, tapering nearly to a point at the other; there was no matter. The wound healed by the first intention, and the patient was soon well again. What is very remarkable about this case is, that the lady has no idea, up to this time, how the glass got into her arm; as she has never met with any accident, or received any injury, by which it could have entered her body anywhere. I requested her to think back, and see if she could not recollect some circumstance that would account for the glass being in her arm; but neither she, nor her husband, nor her parents, nor any of her relations can give me any satisfaction in regard to the case, she declaring that it never entered her body, unless taken with the food into the stomach, and that she has no idea of such an occurrence as that taking place without her knowledge. I could find no cicatrix upon the arm, tha would indicate that the glass even entered there.

This lady, and her husband and relatives, are people of the best standing and are noted for truth and veracity.

Yours, &c.,

H. A. Spencer, M. D.

